# Essential Role of GATA2 in the Negative Regulation of Type 2 Deiodinase Gene by Liganded Thyroid Hormone Receptor β2 in Thyrotroph

**DOI:** 10.1371/journal.pone.0142400

**Published:** 2015-11-16

**Authors:** Hideyuki Matsunaga, Shigekazu Sasaki, Shingo Suzuki, Akio Matsushita, Hirotoshi Nakamura, Hiroko Misawa Nakamura, Naoko Hirahara, Go Kuroda, Hiroyuki Iwaki, Kenji Ohba, Hiroshi Morita, Yutaka Oki, Takafumi Suda

**Affiliations:** 1 Second Division, Department of Internal Medicine, Hamamatsu University School of Medicine, 1-20-1 Handayama, Higashi-ku, Hamamatsu, Shizuoka, 431–3192, Japan; 2 Kuma Hospital, 8-2-35 Shimoyamate-dori, Chuo-ku, Kobe, Hyogo, 650–0011, Japan; 3 Division of Endocrinology, Seirei Hamamatsu General Hospital, 2-12-12 Sumiyoshi, Naka-ku, Hamamatsu, Shizuoka, 430–0906, Japan; 4 Duke-NUS Graduate Medical School Singapore, No 8 College Road, Level 8th, 169857, Singapore; 5 Department of Family and Community Medicine, Hamamatsu University School of Medicine, 1-20-1 Handayama, Higashi-ku, Hamamatsu, Shizuoka, 431–3192, Japan; Hokkaido University, JAPAN

## Abstract

The inhibition of thyrotropin (thyroid stimulating hormone; TSH) by thyroid hormone (T3) and its receptor (TR) is the central mechanism of the hypothalamus-pituitary-thyroid axis. Two transcription factors, GATA2 and Pit-1, determine thyrotroph differentiation and maintain the expression of the β subunit of TSH (TSHβ). We previously reported that T3-dependent repression of the TSHβ gene is mediated by GATA2 but not by the reported negative T3-responsive element (nTRE). In thyrotrophs, T3 also represses mRNA of the type-2 deiodinase (D2) gene, where no nTRE has been identified. Here, the human D2 promoter fused to the CAT or modified Renilla luciferase gene was co-transfected with Pit-1 and/or GATA2 expression plasmids into cell lines including CV1 and thyrotroph-derived TαT1. GATA2 but not Pit-1 activated the D2 promoter. Two GATA responsive elements (GATA-REs) were identified close to cAMP responsive element. The protein kinase A activator, forskolin, synergistically enhanced GATA2-dependent activity. Gel-shift and chromatin immunoprecipitation assays with TαT1 cells indicated that GATA2 binds to these GATA-REs. T3 repressed the GATA2-induced activity of the D2 promoter in the presence of the pituitary-specific TR, TRβ2. The inhibition by T3-bound TRβ2 was dominant over the synergism between GATA2 and forskolin. The D2 promoter is also stimulated by GATA4, the major GATA in cardiomyocytes, and this activity was repressed by T3 in the presence of TRα1. These data indicate that the GATA-induced activity of the D2 promoter is suppressed by T3-bound TRs via a tethering mechanism, as in the case of the TSHβ gene.

## Introduction

Negative feedback regulation in the hypothalamus-pituitary-thyroid (H-P-T) axis is the central mechanism for the homeostasis of thyroid function [1]. In thyrotrophs, intracellular concentration of 3,5,3’-triiodothyronine (T3) determines the inhibition of thyrotropin (thyroid stimulating hormone, TSH) production. Therefore, the conversion of prohormone, thyroxine (T4), to T3 in this cell lineage is a critical step for H-P-T axis [2]. Deiodinases are classified to three isoforms, D1, D2 and D3 [2,3]. Accumulated lines of evidence suggest that D2 is the major determinant of T3 concentration in thyrotrophs [4–11]. In pituitary, only thyrotrophs express two transcription factors, Pit-1 and GATA2, both of which are necessary for their differentiation while somatotrophs express the former but lack the latter [12]. GATA2 may be involved in D2 expression in thyrotroph because D2 activity in thyrotroph-derived TαT1 cells and TSHoma-derived TtT97 cells are much higher than that in somatotroph-derived GH4C1 cells [2,13]. D2 is also expressed in cardiomyocytes, where another GATA family member, GATA4, is expressed [14]. Based on the prediction of several GATA-responsive elements (GATA-REs) in the D2 genes by the computer searches, Dentice et al. [15] compared the activation of the D2 promoters by GATA4 with that of Nkx-2.5, another transcription factor in cardiomyocytes [14]. Although their reporter assay with Hela cells suggested that activation by GATA4 may be much weaker than that by Nkx-2.5, further study should be necessary because the protein level of GATA4 was not verified in their report.

D2 activity in thyrotroph is inhibited by T3 mainly at the transcriptional level [13,16,17]. The best example of the negative regulation by T3 has been described for the gene encoding the β subunit of TSH (TSHβ) [18]. In thyrotrophs, the pituitary specific T3 receptor (TR), TRβ2, is known to mediate its inhibition by T3 [19,20]; however, the downstream mechanism has not been settled [1,21–23]. By analogy with T3-responsive element (TRE) in the genes, which are activated by T3 [24], it has long been postulated that the TSHβ gene may harbor the negative TRE (nTRE) [18,25]. However, this hypothesis was proposed without consideration of GATA2 and Pit-1, both of which are the transcription factors essential for TSHβ expression and thyrotroph differentiation [12]. Indeed, our promoter analysis in the presence of these factors revealed that the nTRE is not necessary for its inhibition by T3-bound TRβ2 [26]. We also found that the major activator for the TSHβ gene is GATA2 while Pit-1 protects the GATA2 function from suppression by the sequence downstream to GATA-REs [27]. The observation that GATA2 Zn-finger domain physically interacts with DNA binding domain (DBD) of TR [26] lead us to the tethering model where TRβ2 interferes with GATA2-induced transactivation in a T3 dependent-manner [1,26].

Because no nTRE has been reported in the D2 gene [8,28], we wanted to know whether our tethering model can be extended to the negative regulation of the D2 gene. Cell-based reconstitution systems were employed because TRβ2 expression is suppressed by thyroid hormones [29] and GATA2 function may be enhanced by thyrotropin-releasing hormone (TRH) [30]. We found that GATA2 but not Pit-1 activates the D2 promoter. The identified GATA-REs are different from those previously predicted by the computer searches [15,31] and exist in the vicinity of the cAMP-responsive element (CRE) [16]. Protein kinase A signaling synergistically enhanced the GATA2-induced transactivation. GATA2-induced activity of the D2 promoter was inhibited by T3-bound TRβ2. When the expression level of GATA4 was adjusted to that of Nkx-2.5, the D2 promoter is also activated robustly by GATA4 and this activity was repressed by T3 in the presence of TRα1, a major TR in cardiomyocytes [32]. Thus, GATAs may meditate the negative regulation of the D2 gene by T3-bound TRs via the tethering mechanism as in the case of the TSHβ gene.

## Materials and Methods

### Plasmid constructions

The firefly luciferase-based reporter gene may be artificially suppressed by T3/TR [1,33]. This possibility was recently reported by Zhang et al. [34]. Thus, we employed a chloramphenicol acetyltransferase (CAT)-based reporter gene. The human D2 promoter encompassing nt. −744/+23 was amplified by polymerase chain reaction (PCR) with forward hDIO2-UME primer (5′-ggggacgcgtctagaattcgaatgtcgcctagctccttcc-3′) and reverse hDIO2-DSX primer (5′-ttttcccgggctcgagcaaagtgcctctctctgcagg-3′). The human TSHβ promoter (nt. −128/+37) in TSHβ-CAT was replaced by this PCR product by digesting with restriction enzymes (MluI and XhoI) to generate hD2-CAT. In this construct, the pUC-derived AP-1-like sequence was deleted because it may also mediate artifactual T3-dependent inhibition [1,33]. hD2-CAT was amplified by PCR with the fixed reverse primer hDIO2-DSX and the various forward primers hDIO2-U1ME (5′-ggggacgcgtctagaattcttttgcattttcttaaataagataat-3′), hDIO2-U2ME (5′-ggggacgcgtctagaattcataatgataatcagaagagagagtgttggccat-3′), hDIO-U2ME (5′-ggggacgcgtctagaattctttcctgaaggctgtcaagggt-3′), hDIO-D4UME (5′-ggggacgcgtctagaattcaaagccctctttctcaatgacg-3′), and hDIO-D5UME (5′-ggggacgcgtctagaattccacttctctattgcagcaattagc-3′) to generate PCR products for Δ1 to Δ5, respectively. The human D2 promoter region encompassing nt. −744/+23 of the hD2 promoter was replaced with these PCR products (Δ1 to Δ5) by digesting with MluI and XhoI. The PCR product for the human D2 gene encompassing nt. −744/+23 was also fused with a modified Renilla luciferase (hRluc)-based reporter system (Promega Corp., Madison, WI, USA). We previously confirmed that hRluc cDNA does not mediate artifactual T3-dependent inhibition [33]. Using a site-directed mutagenesis kit (Stratagene, La Jolla, CA, USA), we mutated u-GATA-RE (M1, RM1), d-GATA-RE (M2, RM2) and both GATA-RE sites (M3, RM3) in hD2-CAT and hD2-hRluc. Expression plasmids for rat TRβ2 (pCMX-rTRβ2), human Pit-1 (pCB6-hPit-1), mouse GATA2 (pcDNA3-mGATA2) [20] and TRH receptor (TRH-R) [30] have been described previously. All subcloning sites and mutated sequences were confirmed by sequencing. Expression plasmids for FLAG-tagged GATA4 and Nkx-2.5 were kindly provided by Dr. Eric N. Olson (University of Texas Southwestern Medical Center, TX, USA).

### Cell culture and transient transfection

Monkey kidney-derived CV1 cells were grown in a monolayer culture at 37°C under CO2/air (1:19) in Dulbecco’s modified Eagle’s medium (DMEM) containing 10% (v/v) fetal calf serum (FCS), penicillin G (100 units/ml), and streptomycin (100 μg/ml). CV1 cells were trypsinized and plated in six-well plates for 24 h prior to transient transfection using the calcium-phosphate technique. Cells at a density of 2×10^5^ cells per well were transfected with 1.0 μg of the hD2-CAT reporter gene, 0.2 μg of pCB6-hPit-1, pcDNA3-mGATA2 and pCMX-rTRβ2 together with 0.9 μg of the β-galactosidase expression vector, pCH111 (a modified version of pCH110, Pharmacia LKB Biotechnology, Piscataway, NJ, USA). The total amount of expression plasmid was adjusted with the empty pCMX vector (3.6 μg of DNA in total per dish). After cells were exposed to calcium phosphate/DNA precipitates for 20 h, the medium was replaced with fresh DMEM containing 10% FCS depleted of thyroid hormone [20] or medium supplemented with T3. Cells were harvested after incubation for an additional 24 h, and CAT activity was measured as described previously [20]. JEG3 cells, choriocarcinoma-derived cell line [35], were cultured in OptiMEM-1 medium (BRL-Gibco, Grand Island, NY, USA) containing 2% FCS, penicillin G (100 units/ml), and streptomycin (100 mg/ml) and transfection with hD2-hRluc and its mutants (RM1, RM2 and RM3) was performed as described previously [33]. GH3 cells, a rat somatolactotroph cell line [36], were kept in DMEM supplemented with 10% FCS, and transfection was performed as described previously [26]. Mouse thyrotroph-derived TαT1 cells [37] (a kind gift from Dr. Pamela Mellon, University of California, CA, USA) were seeded on Matrigel-coated plates (Becton Dickinson Labware, Bedford, MA, USA). The cells were maintained under the same conditions as for CV1 cells. TαT1 cells at a density of 2×10^5^ cells per well were transfected with 4.0 μg of the hD2-hRluc reporter gene and 0.4 μg of pcDNA3-mGATA2, pCB6-hPit-1 and pCMX-rTRβ2 using Lipofectamine2000 reagent (Life Technologies, Carlsbad, CA, USA) according to the manufacturer’s protocol. After incubation for 5 h, the medium was replaced with fresh DMEM containing 5% FCS depleted of thyroid hormones or with DMEM medium supplemented with T3 [26]. CAT and Renilla luciferase activities were normalized for transfection efficiency, as determined by the β-galactosidase assay. We performed transfections with pCMV-CAT (5.0 ng/well) or pGL4.74[hRLuc/TK] (2.0 μg/well), for each reporter assay, the magnitudes of which were adjusted to a value of 100.

### Western blot analysis

To assess the levels of FLAG-tagged GATA2 and FLAG-tagged Nkx-2.5, CV1 cells in a 6 cm dish were transfected with an equal amount (5 μg/dish) of these expression plasmids. After incubation for an additional 24 h, cells were harvested and whole cell extracts were fractionated by sodium dodecyl sulfate polyacrylamide gel electrophoresis (SDS-PAGE) and subjected to western blot analysis with an anti-FLAG antibody (Sigma, St. Louis, MO, USA).

#### Gel-shift assay

A wild-type probe encompassing u- and d-GATA-REs (sense, 5′-tcaagatctttaccaagattaggct-3′; and antisense, 5′-agcctaatcttggtaaagatcttga-3′) was labeled with γ-^32^P-ATP using T4 polynucleotide kinase (Toyobo, Tokyo, Japan). CV1 cells were transfected with pcDNA3-mGATA2 (5 μg per 10 cm dish). After incubation with TRH or tetradecanoylphorbol acetate (TPA), cells were harvested. Nuclear extracts from CV1 cells were prepared as described previously [30]. The γ-^32^P- labeled probes and 2 μg nuclear extract from transfected CV1 cells were incubated for 30 min on ice in 20 μl binding buffer containing 10 mM Tris-HCl (pH 7.6), 50 mM KCl, 0.05 mM EDTA, 2.5 mM MgCl2, 8.5% glycerol, 1 mM dithiothreitol, 0.5 μg/ml poly (dI-dC), 0.1% TritonX-100, and 1 mg/ml nonfat dried milk. DNA–protein complexes were resolved by electrophoresis on 5% polyacrylamide gels at 100 V for 80 min at room temperature. The binding signal was competed by a 50-fold molar excess of the cold competitors: m1 (mutation in u-GATA-RE, sense; 5′-tcaagggctttaccaagattaggct-3′ and antisense; 5′-agcctaatcttggtaaagcccttga-3′), m2 (mutation in d-GATA-RE, sense; 5′-tcaagatctttaccaagggtaggct-3′ and antisense; 5′-agcctacccttggtaaagatcttga-3′) and m3 (mutation in u- and d-GATA-RE, sense; 5′-tcaagggctttaccaagggtaggct-3′ and antisense; 5′-agcctacccttggtaaagcccttga-3′). For the supershift assay, antibodies against GATA2 were added. The gel was dried and labeled bands were visualized using the BAS-1000 autoradiography system (Fuji Film, Tokyo, Japan).

### Chromatin immunoprecipitation (ChIP) assay

Approximately 10^6^ TαT1 cells were grown in 60 mm dishes and the cells were cross-linked with formaldehyde (1% final concentration) for 10 min at room temperature. After cross-linking was terminated by the addition of glycine (0.125 M final concentration), cells were washed twice with ice-cold phosphate-buffered saline, and collected by centrifugation. The cell pellet was resuspended in 200 μl SDS lysis buffer (50 mM Tris-HCl, 10 mM EDTA, 1% SDS, 0.5 mM phenylmethylsulfonyl fluoride, 2 μg/ml leupeptin, 2 μg/ml aprotinin), and incubated for 15 min on ice. Samples were sonicated for 10 sec three times and centrifuged at 14,000 rpm at 4°C. The supernatants were diluted 10-fold with ChIP dilution buffer [50 mM Tris-HCl, 167 mM NaCl, 1.1% Triton X-100, 0.11% sodium deoxycholate (DOC)] supplemented with protease inhibitors. Chromatin solutions (2 ml) were precleared with 60 μl 50% protein G-Sepharose/salmon sperm DNA slurry (Upstate Biotechnology, Lake Placid, NY, USA), and incubated with 4 μl antiserum against GATA2 overnight at 4°C. Immunoprecipitated proteins were recovered with 20 μl 50% protein G-Sepharose/salmon sperm DNA for 2 h and washed with low-salt buffer (50 mM Tris-HCl, 150 mM NaCl, 1 mM EDTA, 1% Triton X-100, 0.1% SDS, 0.1% DOC). Pellets were washed with high-salt buffer (50 mM Tris-HCl, 500 mM NaCl, 1 mM EDTA, 1% Triton X-100, 0.1% SDS, 0.1% DOC), followed by one wash with LiCl wash solution (10 mM Tris-HCl, 250 mM LiCl, 1 mM EDTA, 0.5% Nonidet P-40, 0.5% DOC), and two washes with Tris-EDTA. Protein-DNA complexes were eluted with the elution buffer (10 mM Tris-HCl, 300 mM NaCl, 5 mM EDTA, 0.5% SDS), and cross-linking was reversed by heating at 65°C for 4 h. DNA was extracted with phenol-chloroform-isoamylalcohol (25:24:1) and precipitated with 20 μg of glycogen as a carrier. Samples were dissolved in 20 μl of TE. Using the SYBR Green I kit and a Light Cycler (Roche Diagnostics, Mannheim, Germany), the precipitated DNA was quantified by real-time PCR with primers designed to encompass u- and d-GATA-REs in the human D2 promoter (forward primer: 5′-agtaagccctctttctcaatg-3′, reverse primer: 5′-ttccctggctaattgctg-3′). The thermal cycling conditions were 10 min at 95°C, followed by 40 cycles of 10 sec at 95°C for denaturing, 10 sec at 62°C for annealing, and 7 sec at 72°C for extension. PCR signals were analyzed using Light Cycler software version 3.5 (Roche Diagnostics).

### Statistical analysis

Each CAT or Renilla luciferase reporter assay was performed in duplicate three or more times, and each result was expressed as the mean ± S.E. Significance was examined by ANOVA and Fisher’s protected least significant difference test using Stat View 4.0 software (Abacus Concepts, Berkeley, CA, USA). A value of P<0.05 was considered significant.

## Results

### The human D2 promoter containing nt. −744/+23 is activated by GATA2 and GATA4 but not by Pit-1

**Fig 1:**
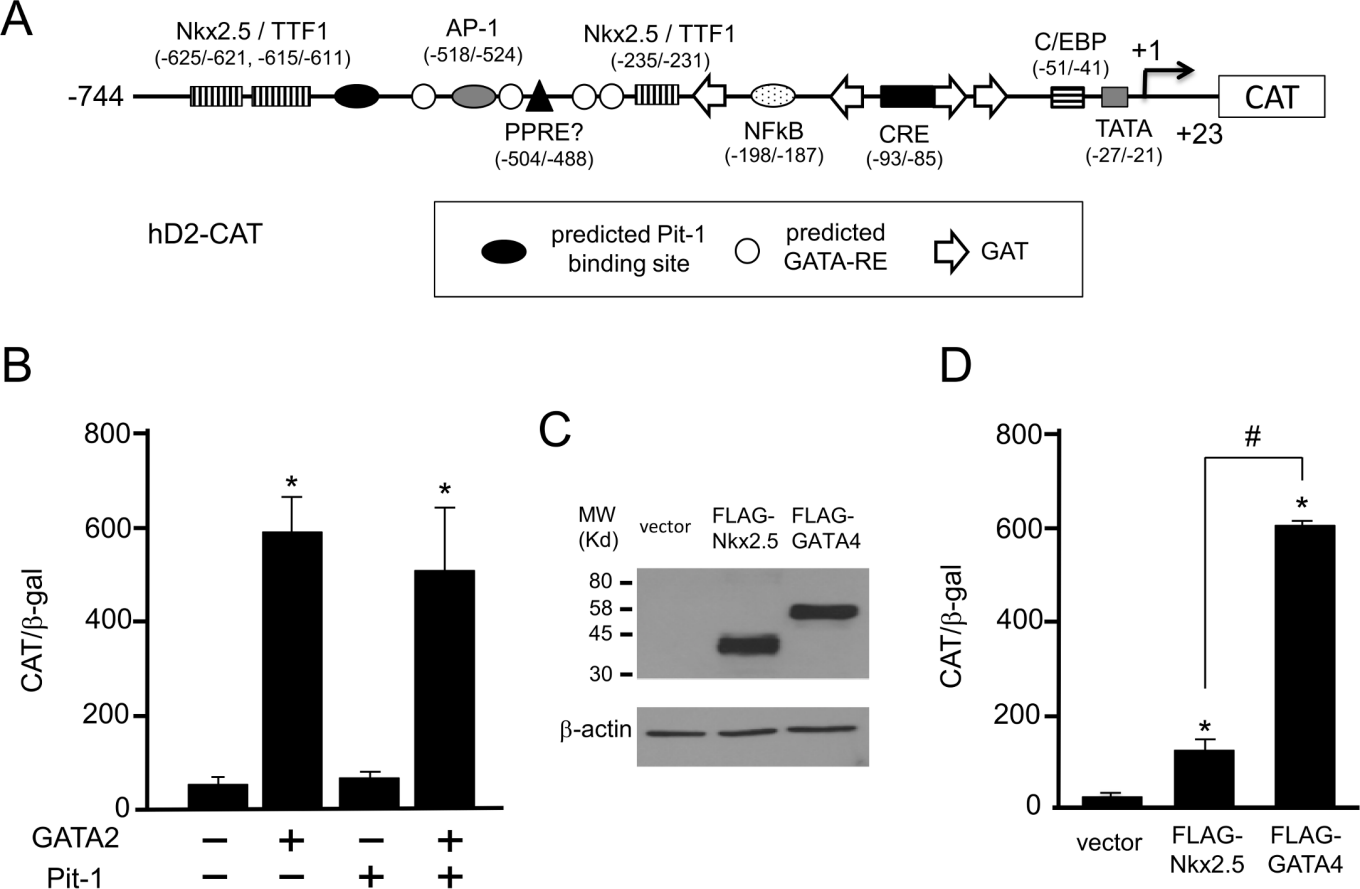
GATA2 and GATA4 but not Pit-1 activate the human D2 promoter. (A) Schematic depiction of hD2-CAT. Various transcription factor recognition sites are indicated: Nkx-2.5/TTF-1, Nkx-2.5-binding site; PPRE?, putative peroxisome proliferator-activated receptor-responsive element. A Pit-1-binding site [38] and the four GATA-REs [15] predicted previously by computer search are indicated as a closed oval and open circles, respectively. The GAT sequences and their inverted sequences (ATC) are indicated as open arrows. The transcription start site is indicated as +1. (B) GATA2 but not Pit-1 transactivates hD2-CAT. Using the calcium phosphate method, CV1 cells at a density of 2×10^5^ cells per well of a six-well plate were transfected with hD2-CAT (2.0 μg) along with the expression plasmid for mouse GATA 2 (pcDNA3-mGATA2) and/or human Pit-1 (pCB6-hPit1). (C) Protein levels of FLAG-tagged GATA4 and FLAG-tagged Nkx-2.5 expression plasmids. CV1 cells in a 6 cm dish were transfected with an equal amount (5 μg/dish) of these expression plasmids. After incubation for 24 h, cells were harvested and subjected to western blot analysis with anti-FLAG antibody (upper panel) and anti-β-actin antibody (lower panel). (D) Activation of the human D2 promoter by FLAG-tagged GATA4 was more potent than that by FLAG-tagged Nkx-2.5. CV1 cells were transfected with hD2-CAT (2.0 μg) along with the equal amounts (0.4 μg/dish) of FLAG-tagged GATA4 or FLAG-tagged Nkx-2.5 expression plasmids. CAT activity for pCMV-CAT (5.0 ng/well) was taken as 100%. Data are expressed as the mean ± S.E. of at least three independent experiments. *, P<0.05 for vector vs. expression plasmids.

Because cDNA sequence of firefly luciferase can mediate artifactual negative regulation by T3 [1,33,34], we employed a CAT-based reporter gene. We fused the human D2 promoter containing nt. −744/+23 to the CAT reporter gene, generating hD2-CAT (Fig 1A). We focused on this promoter region for the following reasons. First, computer search suggested putative GATA-REs [15] and a Pit-1-binding site [38] within this region (Fig 1A). Second, previous deletion analyses of the 6.5 kb promoter region using HeLa cells and rat neonatal cardiomyocytes demonstrated that transcriptional activity of the D2 gene is mainly mediated by a promoter region encompassing nt. −633 to the transcription start site (TSS, nt. +1) [15]. Because these cells express endogenous GATA2 [30] or GATA4 [14], we speculated that, if a functional GATA-RE exists in the human D2 promoter, it will be within this region (nt. −744/+23). Third, since the majority of transcription factor-binding sites previously reported for the D2 gene are included in this region (Fig 1A), the analysis of this region may provide the insight to understand the transcriptional crosstalk among them. hD2-CAT was co-transfected into CV1 cells with the expression plasmids for GATA2 and/or Pit-1. We have confirmed the expression levels of GATA2 and Pit-1 from these plasmids elsewhere [20,26,27,30]. GATA2 rigorously activated hD2-CAT while Pit-1 did not (Fig 1B) and GATA2-induced activity was not affected by the co-expression of Pit-1. These results suggested that a GATA-RE may exist between nt. −744 and +23, and that there is no functional Pit-1 binding site in this promoter region. Next, we decided to re-evaluate the effect by GATA4 on the D2 promoter because the GATA-RE can be recognized by GATA2 as well as GATA4 [39]. Using western blot analysis with an anti-FLAG antibody, we tested expression from FLAG-tagged GATA4 and FLAG-tagged Nkx-2.5 plasmids and confirmed the production of comparable protein levels (Fig 1C). In contrast to a previous report [15], activation of the human D2 promoter by FLAG-tagged GATA4 was more potent than that by FLAG-tagged Nkx-2.5 under our experimental conditions (Fig 1D).

### The newly identified GATA-REs have the DNA sequences different from consensus GATA-RE and exist in the vicinity of the CRE

**Fig 2:**
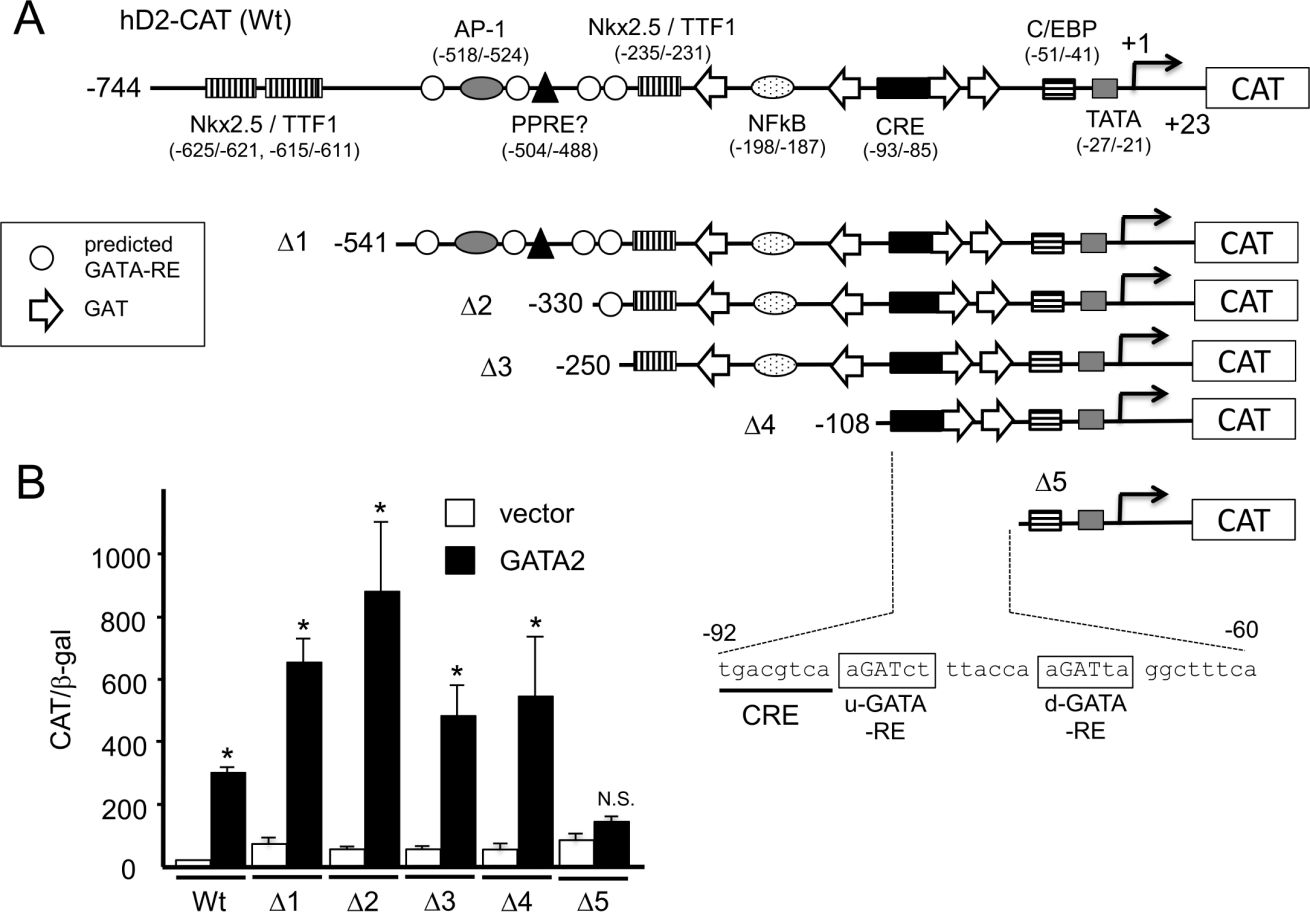
Deletion analysis of the human D2 promoter. (A) A schematic representation of hD2-CAT (wild-type, Wt) and its deletion constructs (Δ1 to Δ5). (B) CV1 cells were transfected with 2.0 μg hD2-CAT (Wt), Δ1 to Δ5 along with 0.4 μg of pcDNA3-mGATA2. Open bars, empty vector; solid bars, pcDNA3-mGATA2. CAT activity for pCMV-CAT (5.0 ng/well) was taken as 100%. Data are expressed as the mean ± S.E. of at least three independent experiments. *, P<0.05 for the empty vector vs. pcDNA3-mGATA2.

**Fig 3:**
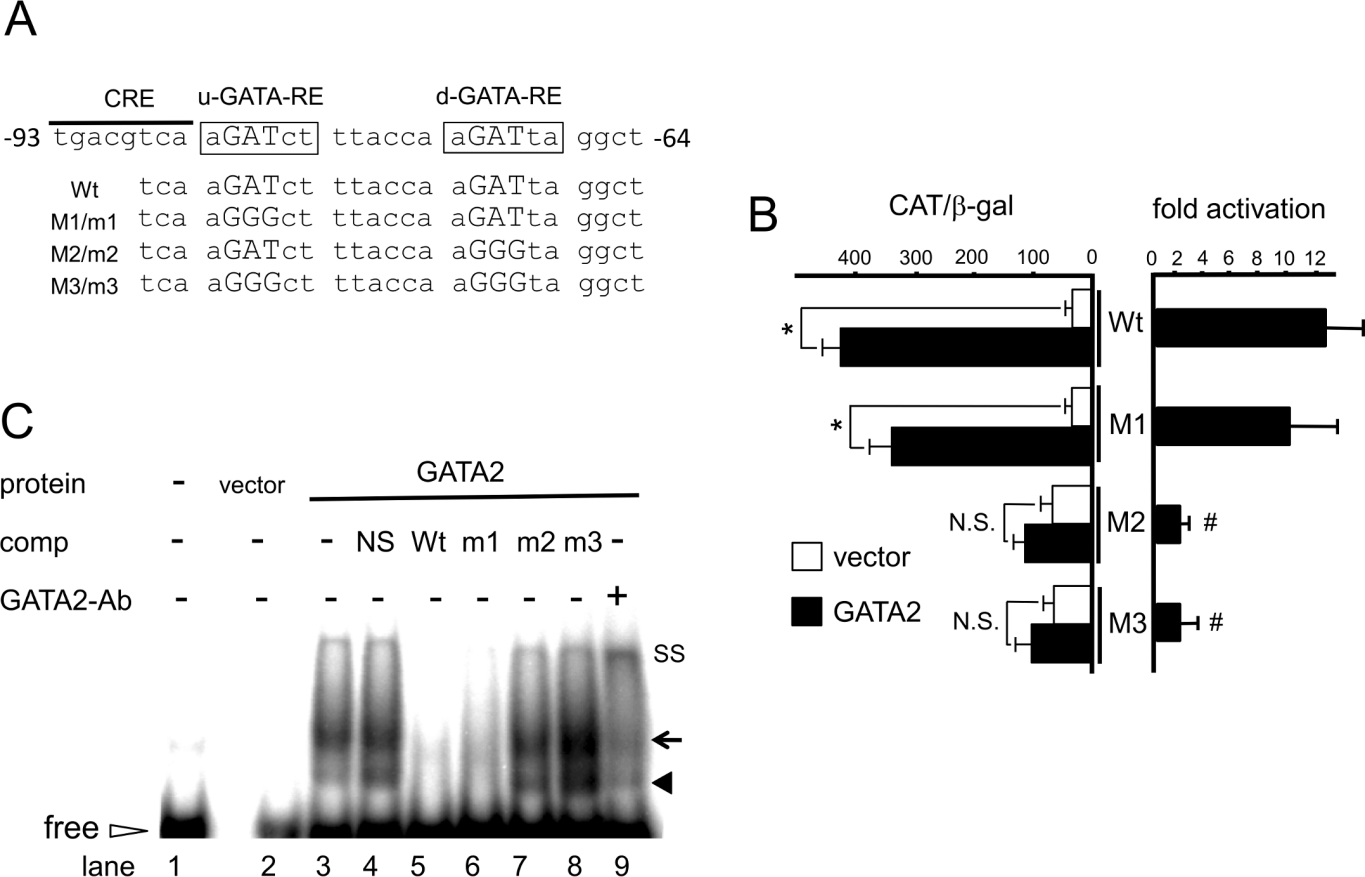
GATA2 recognizes u- and d-GATA-REs in the D2 promoter. (A) A schematic representation of GATA-REs (Wt) and their mutants (M1, M2 and M3 for CAT assay; m1, m2 and m3 for gel shift assay). Two GAT sequences (open arrows) immediately downstream to CRE are designated hereafter as u- and d-GATA-RE. The sequences of wild-type (Wt) and its mutant (M1/m1, M2/m2 and M3/m3) are indicated. (B) Mutation analysis of hD2-CAT. CV1 cells were transfected with 2.0 μg hD2-CAT (Wt) or mutants (M1, M2 and M3; Fig 3A) along with 0.4 μg pcDNA3-mGATA2. Open bars, empty vector; solid bars, pcDNA3-mGATA2. CAT activity for pCMV-CAT (5.0 ng/well) was taken as 100%. Data are expressed as the mean ± S.E. of at least three independent experiments (left panel). *, P<0.05 for the empty vector vs. pcDNA3-mGATA2. To calculate fold activation (right panel), CAT activity with GATA2 was divided by that without GATA2. #, P<0.05 for hD2-CAT (Wt) vs. mutants. N.S., statistically not significant. (C) Gel shift assay using radiolabeled DNA probe containing u- and d-GATA-REs (Wt) or its mutants, m1, m2 and m3 (Fig 3A) with nuclear extract from CV1 cells transfected with pcDNA3-mGATA2. Solid arrowhead, GATA2 monomer; arrow, GATA2 dimer; open arrowhead, free probe; SS, super shift of GATA2 by the anti-GATA2 antibody.

To identify the functional GATA-RE, we performed deletion analysis of hD2-CAT (Fig 2A). Truncation of the reported Pit-1-binding site (Δ1 and Δ2) did not reduce but slightly enhanced transactivation by GATA2 (Fig 2B). Unexpectedly, GATA2 potently activated the deletion construct Δ3, in which all the predicted GATA-REs [15] were deleted. GATA2-induced transactivation was maintained in Δ4 but not Δ5 (Fig 2B), indicative of the existence of a functional GATA-RE between nt. −108 and −60. Although the consensus GATA-RE has been reported as (A/T)GATA(A/G), the GATA family of transcription factors often recognize redundant sequences that contain the sequence GAT [40]. We noticed two such sequences, AGATCT and AGATTA, between nt. −108 and −60 (Fig 2A, open arrows). We mutated these GATs to GGGs, producing three mutant reporter genes, M1, M2 and M3 (Fig 3A). M1 tended to show reduced GATA2-induced activity (Fig 3B) although this was not statistically significant. In contrast, it was severely decreased in M2 and M3, suggesting that these elements, in particular the downstream one, function as GATA-REs. Although the basal promoter activities were slightly increased in M2 and M3, they were not statistically significant (Fig 3B, left panel). We performed gel-shift assays using a ^32^P-labeled DNA probe encompassing these two sequences and nuclear extract from CV1 cells transfected with the GATA2 expression plasmid. As shown in Fig 3C, two bands were detected (lane 3). They were efficiently competed by a 50-fold excess of cold wild-type competitor (lane 5) but not by a nonspecific competitor (lane 4). In addition, these signals were super-shifted by the addition of an anti-GATA2 antibody (lane 9). We generated cold competitor m1, m2 and m3 oligonucleotides (Fig 3A), which correspond to M1, M2 and M3 in the CAT reporter genes (Fig 3B), respectively. Although the binding signals were abolished by a 50-fold excess of m1 (lane 6) and were slightly reduced by that of m2, they were not affected by m3. These observations are in agreement with the results of the reporter assays in Fig 3B. Hereafter, we designate up- and down-stream elements containing the GAT sequence as u-GATA-RE and d-GATA-RE, respectively (Fig 3A).

### The human D2 promoter containing nt. −744/+23 is activated by GATAs endogenously expressed in the choriocarcinoma-derived cell line, JEG3

**Fig 4:**
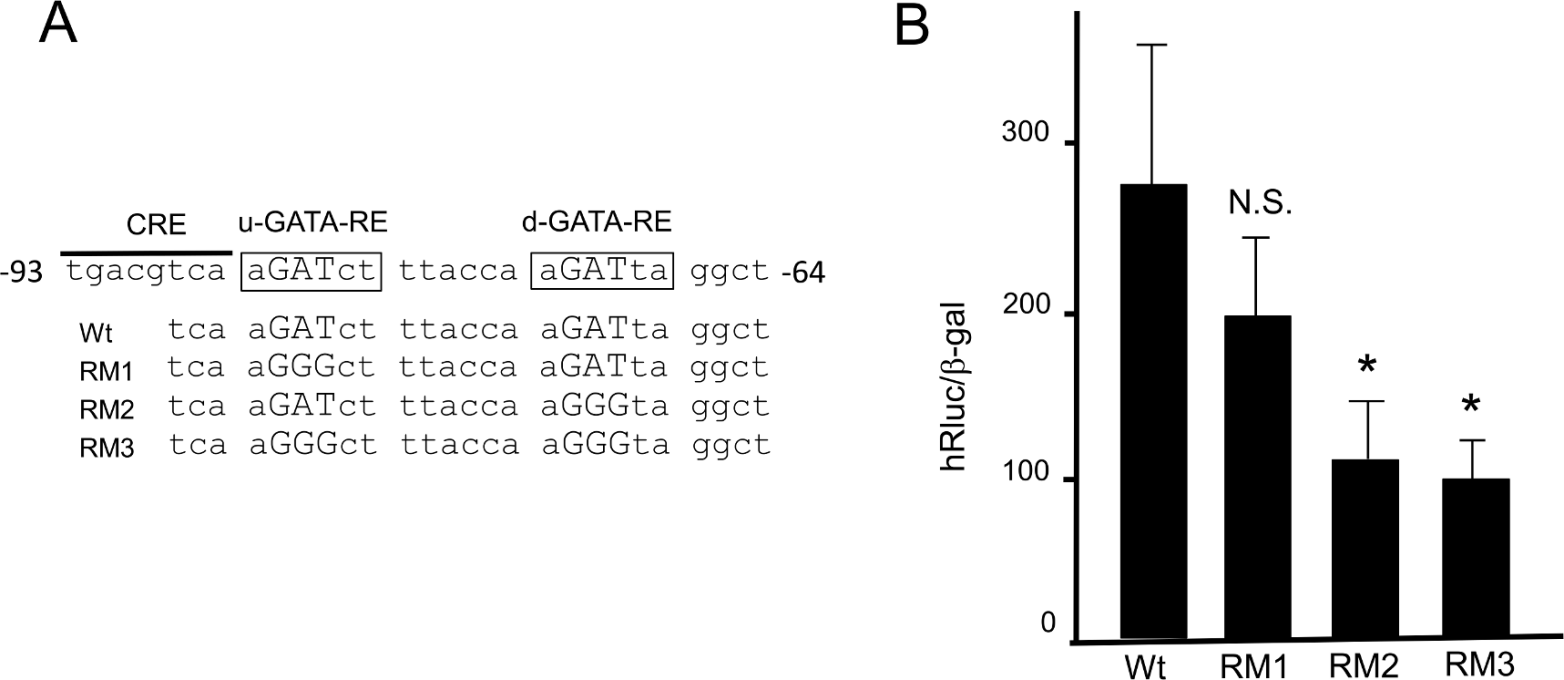
Activation of human D2 promoter by GATA2 or GATA3 endogenously expressed in choriocarcinoma-derived JEG3 cells. (A) A schematic representation of GATA-REs and their mutants (RM1, RM2 and RM3). (B) Using the calcium phosphate method, 2.0 μg hD2-hRluc and its mutants (M1, M2 and M3) were transfected into JEG3 cells. *, P<0.05 for hD2-hRluc (Wt) vs. mutants. The results are means ± S.E. for three independent experiments. pGL4.74[hRLuc/TK] (2.0 μg/well) was used as the inter-assay control and its expression level was adjusted to a value of 100.

We also examined the involvement of u- and d-GATA-REs in the transcriptional control of the D2 gene using the choriocarcinoma-derived cell line, JEG3 [35], which expresses endogenous GATA2 and GATA3 [1,33]. Because transfection efficiency of JEG3 cells is lower than that of CV1 cells [33], we fused the human D2 promoter encompassing nt. −744/+23 (Fig 4A) with a modified Renilla luciferase (hRluc) reporter gene. In addition, we generated its mutants, RM1, RM2 and RM3, which are corresponding to M1/m1, M2/m2 and M3/m3 (Fig 3), respectively. As in the case of CV1 cells (Fig 3B), the D2 promoter activity was significantly reduced in the RM2 and RM3 in this cell line (Fig 4B).

### The PKA but not TRH/PKC pathway synergistically enhances GATA2-induced transactivation of the D2 gene

**Fig 5:**
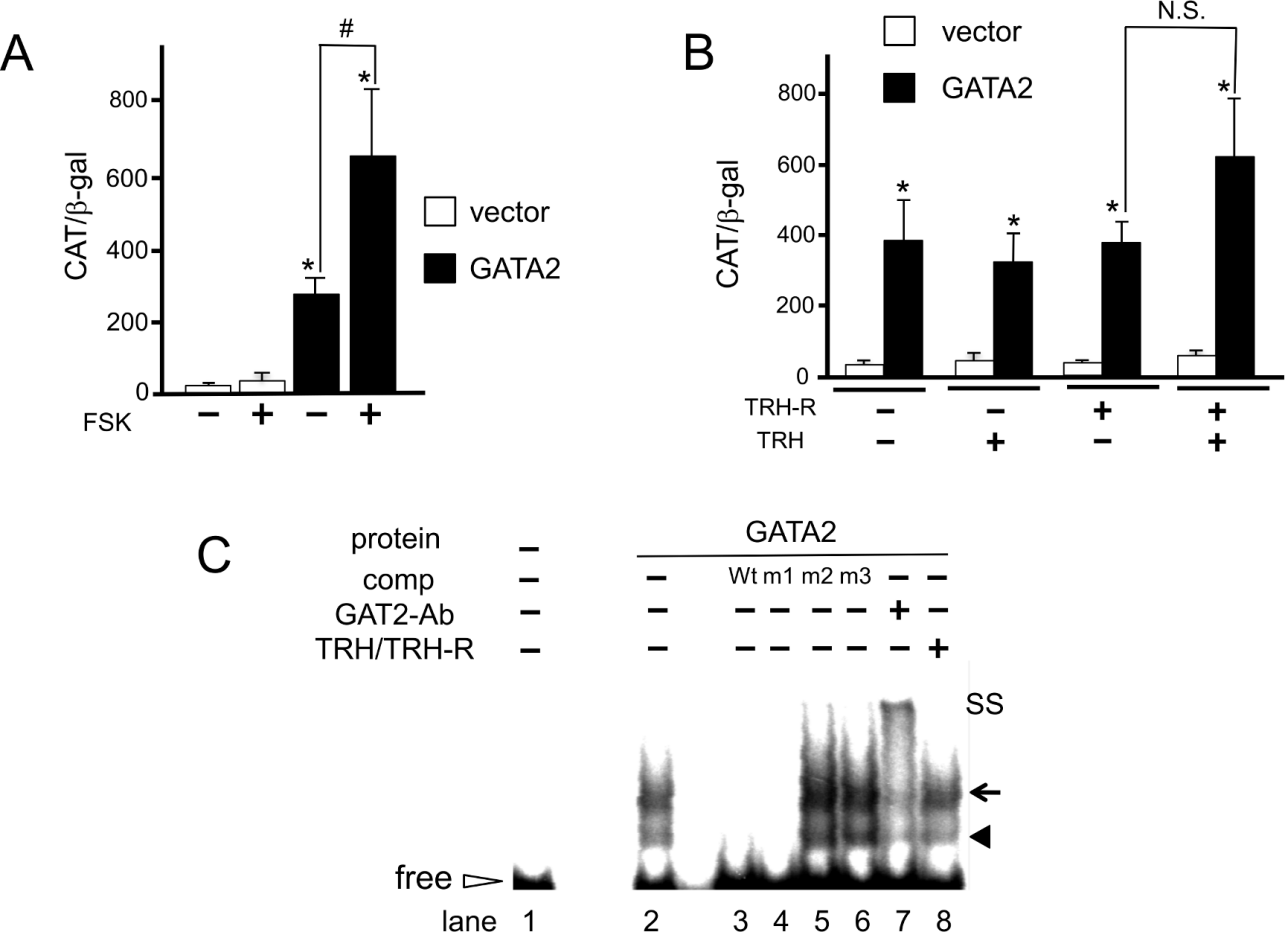
Effects of PKA and PKC signaling on GATA2-induced D2 promoter activity. (A) The hD2 promoter is synergistically activated by GATA2 and PKA signaling. A CAT-reporter assay was performed as described in Fig 1B in the presence or absence of forskolin (FSK) (10 μM). CAT activity for pCMV-CAT (5.0 ng/well) was taken as 100%. Data are expressed as the mean ± S.E. of at least three independent experiments. *, P<0.05 for the empty vector vs. pcDNA3-mGATA2. (B) TRH-R signaling has modest effect on GATA2-induced transcription of the human D2 promoter. After CV1 cells were transfected with 2.0 μg hD2-CAT along with 0.4 μg pcDNA3-mGATA2 and expression plasmid for TRH-R, cells were incubated with 100 nM TRH for an additional 24 h. *, P<0.05 for the empty vector vs. pcDNA3-mGATA2. N.S., statistically not significant. (C) A Gel-shift assay was performed as described in Fig 3C. Solid arrowhead, GATA2 monomer; arrow, GATA2 dimer; open arrowhead, free probe; SS, super-shift of GATA2 by the anti-GATA2 antibody.

Because the GATA-REs exist in the vicinity of the CRE, which mediates PKA signaling and is critical for expression of the D2 gene in several cells and tissues [2,3,8,16], we evaluated the effects of forskolin, a PKA activator. Although the effect of forskolin alone on hD2-CAT was modest and not statistically significant, it was synergistically potentiated by the co-expression of GATA2 (Fig 5A). On the other hand, TRH secreted from the hypothalamic paraventricular nucleus (PVN) is known to stimulate the TSHβ expression via PKC pathway [18]. Indeed, we reported that TRH signaling facilitates the DNA-binding of GATA2, resulting in 3.9 fold potentiation of the GATA2-dependent activation of the TSHβ promoter (nt. -128/+37) fused to CAT reporter gene [30]. Previous study, however, demonstrated that D2 expression in the pituitary is not decreased when the PVN is destroyed [41], suggesting the possibility that GATA2-induced activity of the D2 promoter may be independent from TRH/PKC signaling. Using CV1 cells transfected with the expression plasmids for GATA2 and TRH receptor (TRH-R), we tested the effect of TRH on the activity of the hD2-CAT. As shown in Fig 5B, the increase in GATA2-dependent activation by 100 nM TRH was modest, as evaluated by fold activation (1.6 +/− 0.36), and it was not statistically significant. Using nuclear extracts of CV1 cells that had been co-transfected with expression plasmids for GATA2 and TRH-R, we performed gel-shift assays to examine the effect of TRH signaling on GATA2 binding to u- and d-GATA-Res. As shown in Fig 5C, treatment with 100 nM TRH did not affect the DNA recognition by GATA2. Similar results were observed in the reporter assay and the gel-shift assay when the cells were treated with the PKC activator, TPA (data not shown).

### T3-bound TRs negatively regulate GATA-induced activity of the D2 promoter in CV1 and GH3 cells

**Fig 6:**
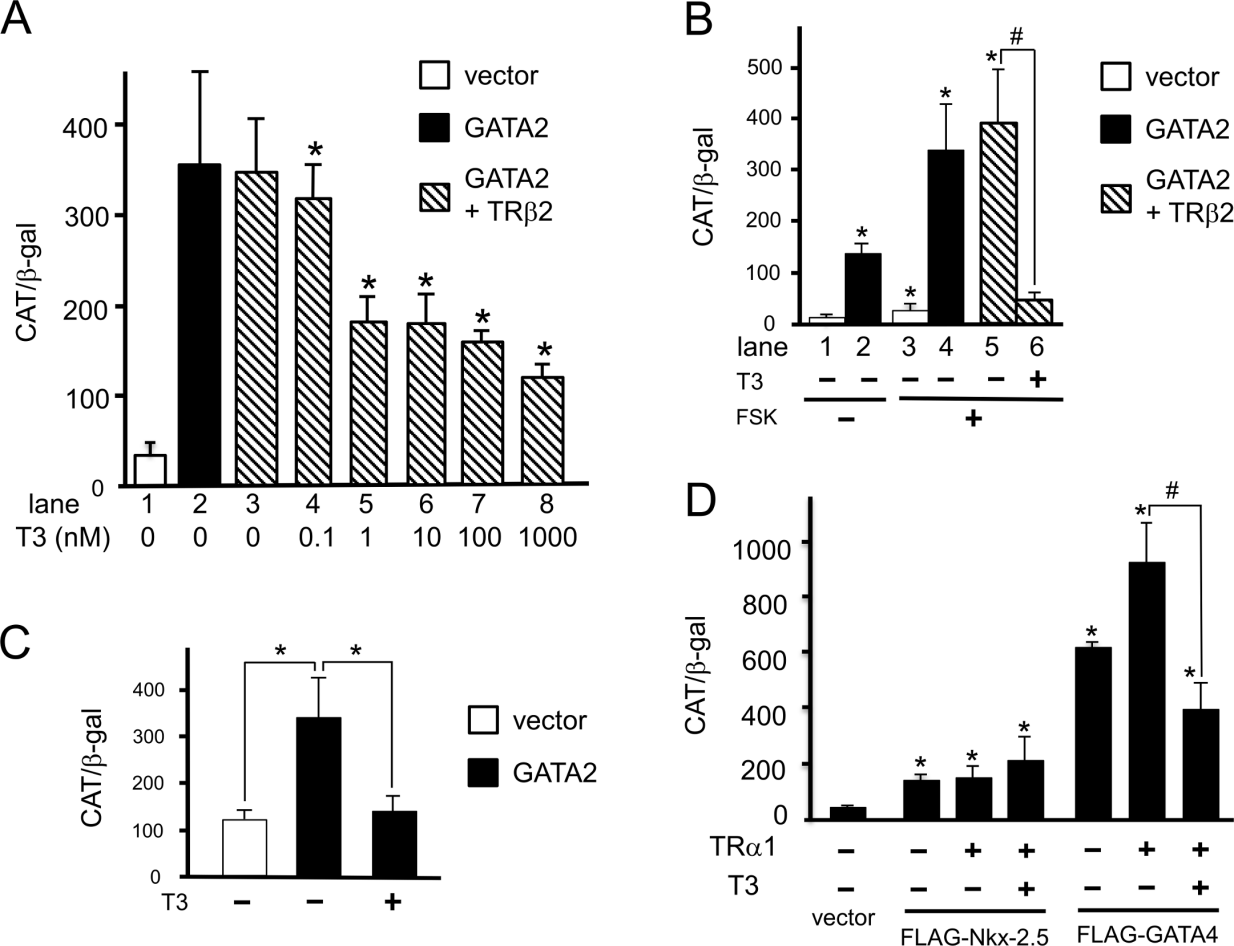
T3-bound TRs negatively regulate the human D2 promoter activity induced by GATAs. (A) Using the calcium phosphate method, 2.0 μg hD2-CAT (wild-type) was transfected into CV1 cells along with the expression plasmids for TRβ2 (0.2 μg) and GATA2 (0.1 μg). *, P<0.05 for T3 (-) vs. T3 (+). (B) The inhibition by T3-bound TRβ2 is dominant over the synergism between GATA2 and PKA signaling. *, P<0.05 for vehicle (lane 1) vs. forskolin (FSK) and/or GATA2. #, P<0.05. (C) Using lipofection, hD2-hRluc (wild-type) was transfected into GH3 cells along with the expression plasmid for GATA2 (0.1 μg) as described in Fig 4. *, P<0.05. (D) Transactivation by GATA4 but not Nkx-2.5 was repressed by T3-bound TRα1. Using the calcium phosphate method, 2.0 μg hD2-CAT (wild-type) was transfected into CV1 cells along with the expression plasmid for TRα1 (0.2 μg), FLAG-tagged Nkx-2.5 (0.1 μg) or FLAG-tagged GATA4 (0.1 μg). *, P<0.05 for the empty vector vs. pcDNA3-mGATA2. #, P<0.05. for T3 (-) vs. T3 (+).

Based on the observation that TRβ2 directly interacts with GATA2 [26], we have proposed a tethering model where TRβ2 interferes with GATA2-induced transactivation of the TSHβ gene in a T3-dependent manner [1,26,30]. As in the case of the TSHβ gene [1], the D2 gene in thyrotrophs has been reported to be repressed by T3 [7,8,13,17,42]. We tested here whether T3-bound TRβ2 may inhibit GATA2-dependent activity of the D2 promoter. As expected, the GATA2-induced activity of hD2-CAT was inhibited by physiological concentrations of T3 in the presence of TRβ2 (Fig 6A, lanes 3–8). The synergistic activation of hD2-CAT by GATA2 and forskolin (Fig 6B, lane 4) was also suppressed by T3-bound TRβ2 (lanes 5 and 6). As somatolactotroph-derived GH3 cells [36] express endogenous TRβ2 and Pit-1 but not GATA2 [26], these cells transfected with GATA2 enabled us to mimic transcriptional regulation in thyrotrophs [12]. We also confirmed previously that hRluc cDNA does not mediate artifactual inhibition by T3 [33]. As shown in Fig 6C, T3 treatment inhibited the activity of hD2-hRluc induced by GATA2 to basal levels. Because T3 strongly represses expression of the D2 mRNA in rat cardiomyocytes [43], we tested the effect of T3 on the GATA4-induced activity of the D2 promoter in the presence of TRα1, which is the major TR in cardiac tissue [32]. As shown in Fig 6D, transactivation by GATA4 was also inhibited by T3-bound TRα1. By contrast, Nkx-2.5-induced transactivation was not affected by T3-bound TRα1, suggesting that GATA4 but not Nkx-2.5 specifically mediates the negative regulation by T3. We have previously confirmed the expression levels of TRβ2 and TRα1 elsewhere [20,26,30,32].

### GATA2-induced activity of the D2 promoter containing nt. −744/+23 is repressed by T3-bound TRβ2 in the thyrotroph cell line, TαT1

**Fig 7:**
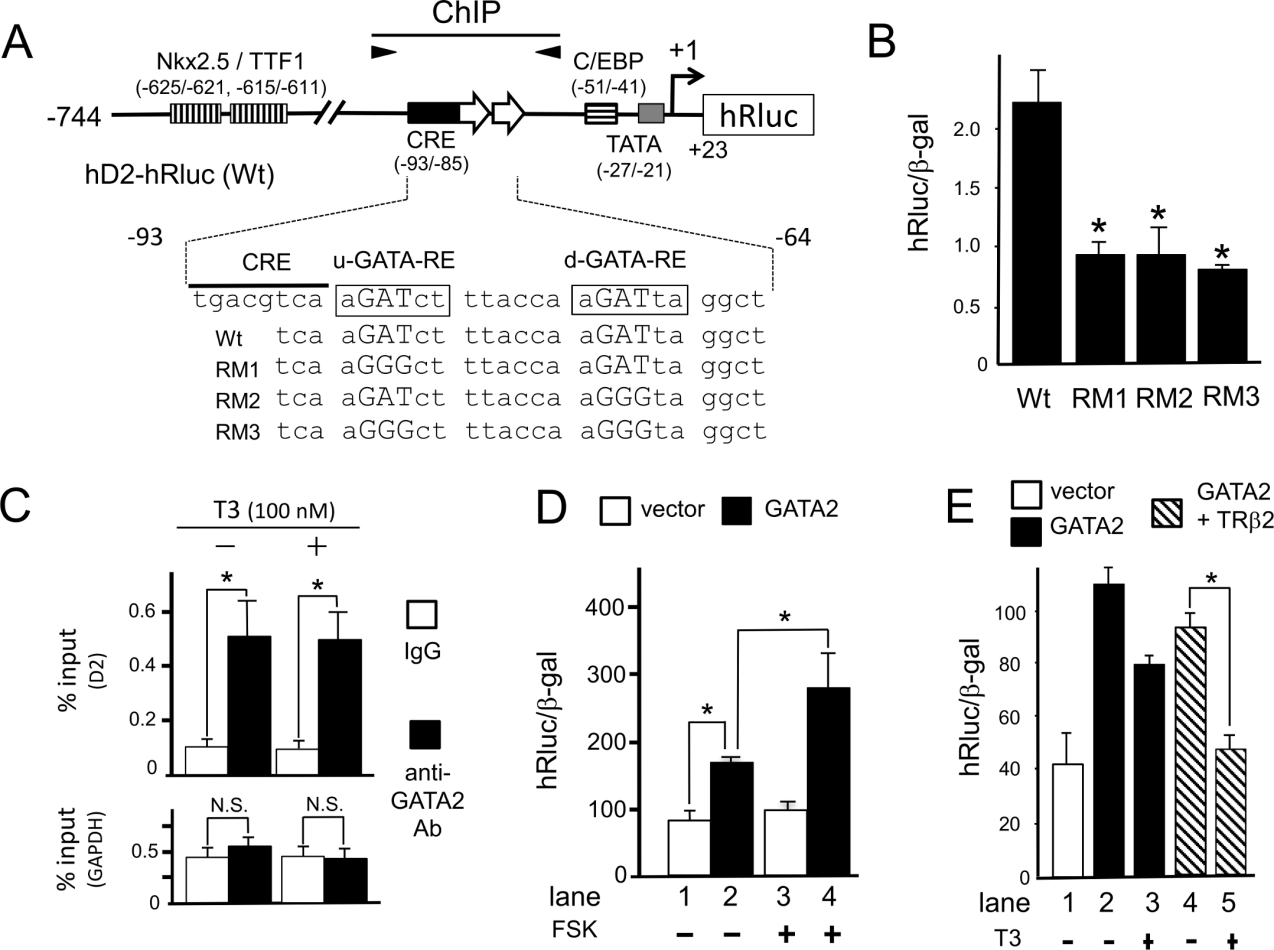
Involvement of GATA2 in T3 negative regulation in the thyrotroph cell line, TαT1. (A) A schematic representation of hD2-hRluc (wild-type, Wt) and its mutants. (B) The hD2 promoter is activated by endogenous GATA2 via u- and d-GATA-REs. Using the lipofection method, 2.0 μg hD2-hRluc and its mutants (RM1, RM2 and RM3) were transfected into TαT1 cells. *, P<0.05 for hD2-hRluc (Wt) vs. mutants. (C) In the absence or presence of 100 nM T3, ChIP assay was performed in TαT1 cells using an antibody against GATA2 or control mouse IgG. Immuno-precipitated chromatin fragments were amplified by PCR with primers for the mouse promoter. The positions of primers in the mouse D2 gene that correspond to the position of the human D2 gene are indicated by closed arrowheads in (A). Left panel, mouse D2 gene; right panel, mouse glyceraldehyde 3-phosphate dehydrogenase (GAPDH) gene. The signals were measured by quantitative real-time PCR. The values are expressed as percentages relative to the levels with anti-GATA2 antibody at 1 h. *, P<0.05. N.S., not significant. (D) After TαT1 cells were transfected with hD2-hRluc along with or without pcDNA3-mGATA2, the cells were incubated in the presence or absence of 10 μM forskolin for an additional 24 h. *, P<0.05. pGL4.74 [hRLuc/TK] (2.0 μg/well) was used as the inter-assay control and its expression level was adjusted to a value of 100. (E) After TαT1 cells were transfected with hD2-hRluc along with or without pcDNA3-mGATA2 and/or pCMX-rTR2, the cells were incubated in the presence or absence of 1 μM T3 for an additional 24 h. *, P<0.05. The results are means ± S.E. for three independent experiments. pGL4.74 [hRLuc/TK] was used as the inter-assay control and its expression level was adjusted to a value of 100.

According to Christoffolete et al. [13], T3 represses transcription of the D2 gene in the thyrotroph cell line, TαT1 [37]. Using this cell line, we tested the effect of mutations in the u- and d-GATA-REs in hD2-hRluc (Fig 7A). As in CV1 cells (Fig 3) and JEG3 cells (Fig 4), mutation of d-GATA-RE reduced the activity of hD2-hRluc (Fig 7B). Unexpectedly, mutation of u-GATA-RE also significantly decreased the activity of the D2 promoter. We performed chromatin immunoprecipitation (ChIP) assays using specific primers encompassing the u- and d-GATA-REs (Fig 7A) and detected in vivo binding of GATA2 with this region (Fig 7C). GATA2 binding was not affected by treatment with 100 nM T3. Because the expression level of endogenous GATA2 in TαT1 cells is modest and comparable to that in HeLa cells [30], we evaluated the effect of over-expression of GATA2 on the D2 promoter in this cell line. As shown in Fig 7D (lanes 1 and 2), the co-transfection of GATA2 increased the activity of the D2 promoter, suggesting that the level of GATA2 is the limiting factor for expression of the D2 gene. Whereas forskolin treatment exhibited minimal effect on the D2 promoter (lanes 1 and 3), it enhanced the GATA2-induced activation (lanes 2 and 4). The synergism between GATA2 and forskolin is consistent with the finding in CV1 cells (Fig 5C). As shown in Fig 7E, T3 treatment tended to reduce the GATA2-induced activity of the D2 promoter, although it was not statistically significant. When TαT1 cells were co-transfected with the expression plasmids for both GATA2 and TRβ2, we detected greater repression by T3, which was statistically significant.

## Discussion

**Fig 8:**
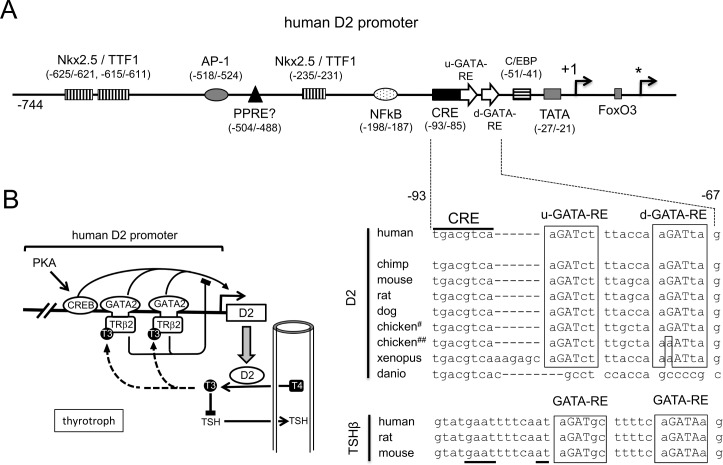
(A) Schematic depiction of the human D2 promoter and difference in the GATA-RE sequences between the D2 gene and TSHβ gene. The promoter structure and the position of the TSS (+1) are based on the reviews by Bianco et al. [8] and Gereben et al. [16]; however, Dentice et al. [66] later reported that FoxO3 activates transcription from another downstream TSS (*). The DNA sequences of u- and d-GATA-REs are conserved among species except for zebra fish (Danio rerio). While one report (Accession AB307676) suggests d-GATA-RE is conserved in chicken (#), another does not (##) [16]. The Pit-1-biding sequence in the TSHβ gene was underlined. (B) Tethering model of T3-dependent negative regulation of the human D2 gene in thyrotrophs. The Zn-finger domain of GATA2 associates with TRβ2-DBD via a protein–protein interaction. In the presence of T3, TRβ2 interferes with the transactivation function of GATA2. The current study predicts a similar molecular mechanism in the negative regulation of the D2 gene by T3 in cardiomyocytes. The inhibition by T3 is dominant over the synergism between GATA2 and PKA signaling via CREB. After pre-existing D2 enzyme converts T4 to T3, the influence of de novo generated T3 is blunted owing to the reduction of D2 expression by T3. Thus, the T3 produced by D2 in thyrotrophs may reflect the real-time level of T4, which is taken up by thyrotrophs from circulating blood.

Here, we showed that the D2 promoter is activated by GATA2, which is one of the determinants for the thyrotroph differentiation [12], via newly identified GATA-REs (Fig 8A). As shown in Fig 1D, this promoter was also activated by GATA4. In contrast to the Nkx-2.5-binding sites [15], u- and d-GATA-REs are evolutionally conserved (Fig 8A) [16]. As shown in Figs 3B, 4B and 7B, physiological relevance of them may be cell-specific. Interestingly, previous studies of the D2 promoter [13,15,44–46] often employed cells that endogenously express GATA family members, i.e., JEG3 [47], HeLa [30,48], MSTO-211H [49], TαT1 [30], TtT97 [50] and rat neonatal cardiomyocytes [14]. Pit-1 is another determinant for the thyrotroph differentiation [12]. Although a Pit-1 binding site was predicted in the human D2 promoter (Fig 1A) [38], it is not conserved in the mouse D2 gene [31] and the result in Fig 1B denies its function.

It is known that PKA stimulates the D2 promoter via its CRE [16,45]. Here, we found that PKA signaling is strongly synergized by GATA2 (Figs 5A and 7C). It is known that CRE-binding protein (CREB) associates and synergizes with GATA3, of which amino acid sequence is most similar to that of GATA2 among the GATAs [51]. Because the CRE locates immediately upstream of the GATA-REs (Fig 8A), CREB may interact with GATAs. The importance of this CRE was previously demonstrated by fusion with a heterologous thymidine kinase promoter [52]. Interestingly, the DNA sequence transferred as the CRE also contained both u- and d-GATA-REs and was assayed with JEG3 cells, which are know to express GATA2 [47]. By contrast, it was reported that cAMP agonists have only a modest effect on D2 expression in the somatotroph cell lines [45], where GATA2 is expected to be absent [12]. We reported previously that TRH signaling from the hypothalamic PVN facilitates the DNA-binding of GATA2, resulting in 3.9 fold activation of the TSHβ promoter (nt. -128/+37) [30]. If TRH signaling can also enhance the GATA2-dependent D2 expression, then elevated T3 production may offset TRH-induced TSHβ expression. Unexpectedly, however, the impact of TRH signaling on the D2 gene was modest (1.6 +/− 0.36 fold) and not statistically significant (Fig 5B). This observation is supported by the previous report that D2 expression in the pituitary was not decreased even when the PVN was destroyed [41]. As shown in Fig 5C, TRH signaling did not facilitate the DNA binding of GATA2. Our findings in Fig 5B and 5C may be explained by the reports that the nucleotides flanking the core GAT sequence (Fig 8A) profoundly modulate the effect of phosphorylation signal on the transactivation by GATAs [53,54].

Although no nTRE has been identified [8,28], T3-bound TRs inhibit the D2 promoter (nt. -744/+23) in the presence of GATAs (Fig 6). The observation that T3-bound TRα1 does not repress Nkx-2.5-induced transactivation (Fig 6D) again excludes the presence of the cryptic nTRE, via which T3-bound TR can repress global activity of this promoter sequence. Several nTREs have been postulated [21–23]. However, majority of them are proposed without consideration of the cell-specific transcription factors [1] and lack the validation by mutation analysis in reporter assay. For example, our deletion study in the presence of GATA2 and Pit-1 revealed that the nTRE reported in the human TSHβ gene [18,25] is not necessary for its inhibition by T3 [26]. Although other TR-binding sequences were suggested in the mouse TSHβ gene using ChIP assay [55], their functions remain to be verified by the mutation analysis. In the MYH7/MyHC-I gene, nTREs have also been postulated [56,57]; however, we [32] and recently other groups [34] reported that T3-induced inhibition is independent from them. Thus, it may be necessary to reconsider the existence of so-called nTRE [1,21–23].

In the context of the TSHβ gene, our co-immunoprecipitation and glutathione-S-transferase pull down assays demonstrated the protein-protein interaction of TRβ2 DBD with GATA2 Zn-finger domain [26]. GATA2-TRβ2 complex was confirmed using GATA-RE DNA conjugated with magnetic beads [26]. Based on these findings, we proposed the tethering model whereby T3-bound TRβ2 inhibits the GATA2-induced transactivation [1]. The results in Fig 6 strongly suggest the similar mechanism in the D2 gene (Fig 8B). Consistently, it was reported that hypothyroidism induces D2 mRNA in rodent heart [43], where both TRs and GATA4 are expressed [14,32]. Corticotroph expresses TR [9,58] but not GATA2 [12]. As expected, repression of D2 expression by T3 is negligible in the corticotroph cell line, AtT20 [59]. Our view may also explain why repression by T3-bound TRβ2 is dominant over the synergism between GATA2 and PKA signaling (Fig 6B). It should be noted, however, that DNA recognition by GATA2 is not affected by T3 (Fig 7C) and that the interaction of TRs with GATAs is independent from T3 [26]. Thus, it is still unknown how TR attenuates RNA polymerase II activity in a T3-dependent manner [1]. In addition, our model does not exclude the GATA-independent mechanism(s) for the inhibition of the D2 gene. As shown in Fig 8A, GATA-REs are not conserved in the D2 promoter of zebra fish (danio rerio); however, T4 represses D2 expression in its pituitary [42]. The mechanism independent from GATA2 was also reported in somatotrophs [60,61] although D2 expression in them may be lower than that in thyrotrophs [2,13].

The physiological relevance of T3-induced repression of D2 in thyrotrophs has not been fully clarified [13,62,63]. The following speculation is possible. First, our model (Fig 8B) predicts that after pre-existing D2 enzyme converts T4 to T3 [2], the influence of de novo generated T3 will be blunted owing to the reduction of D2 mRNA levels by T3. Thus, T3 produced by D2 in thyrotrophs may mainly reflect the real-time T4 level that is up-taken by thyrotrophs from circulating blood. Several lines of evidence support this. For example, the ubiquitin-proteasome system degrades D2 enzyme with short half life (~40 min) in TαT1 cells [13]. It also quickly degrades GATA2 protein (half life = 27.7 min) in murine leukemia-derived cells [64]. Moreover, T3 strongly decreases TRβ2 mRNA level in rat anterior pituitary within 8 hours [29]. Second, T3-induced reduction of D2 expression may ameliorate the abrupt reduction of TSH by T3. Recent curve fitting analysis with large human population revealed that reduction of TSH by free T4 in the range of euthyroid is milder than those of hypothyroidism and hyperthyroidism [65]. Finally, down-regulation of D2 expression may protect thyrotrophs from the cell death in severe hyperthyroidism [42]. However, further investigation is required because the GATA2-dependent transactivation of the D2 gene (Fig 5) and that of TSHβ gene [1,30] may be differentially potentiated by PKA and PKC/TRH signaling, respectively. We are only just beginning to unravel some of complexities involved in the H-P-T axis.
